# What's in a mate? Social pairing decisions and spatial cognitive ability in food-caching mountain chickadees

**DOI:** 10.1098/rspb.2023.1073

**Published:** 2023-09-13

**Authors:** Carrie L. Branch, Joseph F. Welklin, Benjamin R. Sonnenberg, Lauren M. Benedict, Virginia K. Heinen, Angela M. Pitera, Eli S. Bridge, Vladimir V. Pravosudov

**Affiliations:** ^1^ Department of Psychology, The University of Western Ontario, London, Ontario, Canada; ^2^ Department of Biology, University of Nevada, Reno, Reno, NV, USA; ^3^ Ecology, Evolution, and Conservation Biology Graduate Program, University of Nevada, Reno, Reno, NV, USA; ^4^ Department of Biology, University of Oklahoma, Norman, OK, USA

**Keywords:** assortative mating, social mate choice, spatial cognition, chickadees, environmental variation

## Abstract

While researchers have investigated mating decisions for decades, gaps remain in our understanding of how behaviour influences social mate choice. We compared spatial cognitive performance and food caching propensity within social pairs of mountain chickadees inhabiting differentially harsh winter climates to understand how these measures contribute to social mate choice. Chickadees rely on specialized spatial cognitive abilities to recover food stores and survive harsh winters, and females can discriminate among males with varying spatial cognition. Because spatial cognition and caching propensity are critical for survival and likely heritable, pairing with a mate with such enhanced traits may provide indirect benefits to offspring. Comparing the behaviour of social mates, we found that spatial cognitive performance approached a significant correlation within pairs at low, but not at high elevation. We found no correlation within pairs in spatial reversal cognitive performance at either elevation; however, females at high elevation tended to perform better than their social mates. Finally, we found that caching propensity correlated within pairs at low, while males cached significantly more food than their social mates at high elevations. These results suggest that cognition and caching propensity may influence social mating decisions, but only in certain environments and for some aspects of cognition.

## 1. Introduction

Mate choice is a critically important decision for anisogamous species; however, mating systems and the associated consequences of mating decisions vary widely [1]. In the case of socially monogamous species, males and females choose both social and genetic mates, prioritizing direct benefits from their social mate and seeking extra-pair copulations (EPCs) to enhance indirect fitness [2]. Both strategies are common in avian species; with 75% of bird species exhibiting EPCs [3] and 80% of bird species forming social bonds with biparental care [4]. EPCs are often cryptic but significantly contribute to an individual's lifetime reproductive success (e.g. [5]), therefore, genetic mate choice has received a lot of research attention. However, social mates often provision young regardless of genetic paternity [6,7], so the selection of a social mate is expected to have profound reproductive consequences. Social mate choice in the form of positive assortment via morphological characteristics, such as large individuals pairing with large individuals and small individuals pairing with small individuals, has been well-described; however, we know much less about social mating decisions based on behaviour (e.g. [8–10]).

Assortative mating based on behaviour is often described as ‘incidental assortment', where individuals find themselves spatially or temporally segregated, resulting in passive assortment as opposed to ‘choice-driven’, active assortment (e.g. [9,11–14]). Among behavioural traits, cognitive abilities vary between individuals in a breeding population [15,16], providing the opportunity to test for mate choice-driven assortativity of behaviour based on differences in cognition, either by direct observation [17] or indirectly via secondary sexual traits [18]. Historically, it has been assumed that enhanced cognitive abilities are generally beneficial and should improve survival; however, neural substrates supporting cognitive abilities are energetically expensive, resulting in trade-offs [19–21] that generate variation available for sexual selection. Recent research shows that natural selection acts on the spatial cognitive abilities of food-caching birds that rely on relocating food stores to survive harsh winter conditions [22]. Furthermore, a genome-wide association study suggests that variation in the genome provides the foundation for hippocampal neuron growth and development related to individual differences in spatial cognitive abilities [23]. Given that environmental and genetic variation shape spatial cognitive abilities, and these abilities are critical for survival in food-caching birds [24], cognitive abilities should be important in both genetic and social mate choice decisions when individuals are seeking the highest-quality mates.

While spatial cognitive abilities are required to recover food caches, the ability to quickly extinguish one response and learn a new one may allow animals to better track rapidly changing environments [15,25–28]. Such ability to learn changing associations is defined as cognitive flexibility and is traditionally measured using reversal associative learning tasks [15,25–27]. In a single reversal learning task, an animal is expected to inhibit the response to a previously learned association to learn new associations using familiar cues [26–28]. Such inhibition allows faster learning of new or changing associations, which we have argued may result in a trade-off between initial spatial memory load and ability to inhibit such responses [27]. Indeed, we have previously shown that birds inhabiting harsher, high elevations outperform low-elevation birds on the spatial learning and memory task, while low-elevation birds outperform high-elevation birds on a single reversal learning task [29]. In addition, individual performance on the single and serial reversal tasks correlate, while performance on serial reversal and spatial learning and memory task do not, suggesting these cognitive tasks tap into different cognitive processes [28].

Furthermore, propensity to cache food and the amount of food stored are also likely critical for surviving the winter [24]. Among many scatter-hoarding species, the drive to store food is thought to be primarily innate [30], as individuals will cache when food is provided ad libitum in the laboratory and some will cache until available resources are depleted [24,31,32]. Indirect evidence suggests that baseline between-individual variation in caching propensity is heritable [33–35] and repeatable across years despite substantial variation in annual climate [36], suggesting that caching propensity may be relevant for mate choice decisions. Individuals that cache more and have better cognitive abilities to recover those caches are likely to be in better condition coming into the breeding season and therefore may be able to devote more time and energy to provisioning young. Considering that both spatial abilities and caching propensity are likely heritable [23,33–35], selecting mates with better spatial cognition and higher caching propensity may provide indirect genetic benefits to offspring, resulting in increased fitness.

Using ‘smart’ feeder arrays equipped with radio frequency identification (RFID) technology, we compared performance within pairs of mountain chickadees (*Poecile gambeli*) on two cognitive tasks, a spatial learning and memory task and a single reversal learning task. We also compared food caching propensity within social pairs of mountain chickadees (*Poecile gambeli*) to understand if these behaviours are related to social mate choice. Mountain chickadees are non-migratory, food-caching birds that inhabit the montane regions of western North America [37]. These birds store tens of thousands of food items throughout their territories when food is abundant and rely on specialized spatial cognitive abilities to recover those food stores and survive the winter, when food is scarce [22,24,38]. Previous research shows that males and females do not differ in spatial cognitive abilities at the population level, suggesting that natural selection pressures are similar for both sexes [39]. However, female mountain chickadees appear to be able to discriminate among males of differing cognitive abilities. We have shown that females increase their reproductive investment by laying more eggs when breeding conditions are good and their social mate has superior spatial cognitive abilities [40,41]. That said, it is unclear whether females can directly assess males' cognitive abilities by observing successful cache retrieval (e.g. [17]), or if they assess males indirectly via secondary sexual traits. Either way, we do not know if females and males actively choose social mates based on cognitive abilities.

If mountain chickadees choose social mates based on cognitive ability, two possible relationships may emerge: (i) a positive correlation between male and female cognitive performance and caching propensity if the higher-performing males and higher-performing females pair, leading to assortative mating for cognition, or (ii) females may pair with males that perform better on the spatial cognitive task and cache more than themselves [23,24,33,34], leading to a within-pair difference with males performing better than their social mate. In addition, social pairings may change from year to year, either due to death or divorce (both mates detected and paired with other birds). If divorce results from either bird seeking a new mate with better cognitive abilities and caching propensity, we would predict that new mates resulting from divorce would have better spatial cognitive abilities and higher caching propensity than their previous mate, while we should see no difference among individuals that have repaired due to their partners’ death.

## 2. Material and methods

### (a) Study system and site

We investigated the relationship between spatial cognitive ability and social mating choices at our long-term field site in northern California, Sagehen Experimental Forest, USA (Sagehen Creek Field Station, University of California Berkeley, approximately 14.5 km north of Truckee, CA). We have monitored breeding in nest-boxes since 2013 and banded birds at established feeders since 2014 across two elevation sites, referred to herein as low (1900 m) and high (2400 m) elevations. We capture and band birds annually across nine feeders at low elevation and six feeders at high elevation. During autumn trapping and summer breeding, birds are trapped via mist net or by hand in nest-boxes, respectively. Upon capture birds are aged, a blood sample (approx. 100 µl) is taken for genomic analyses, flattened wing length is measured, and each bird is fitted with a uniquely identifying colour combination, including a passive integrated transponder (PIT) tag and one or two other colour bands. PIT tags are then used to track performance during spatial cognitive testing (e.g. [16]) and confirm bird identification using RFID antennas placed in feeders and nest-box entrances.

### (b) Cognitive testing

**Figure 1.**
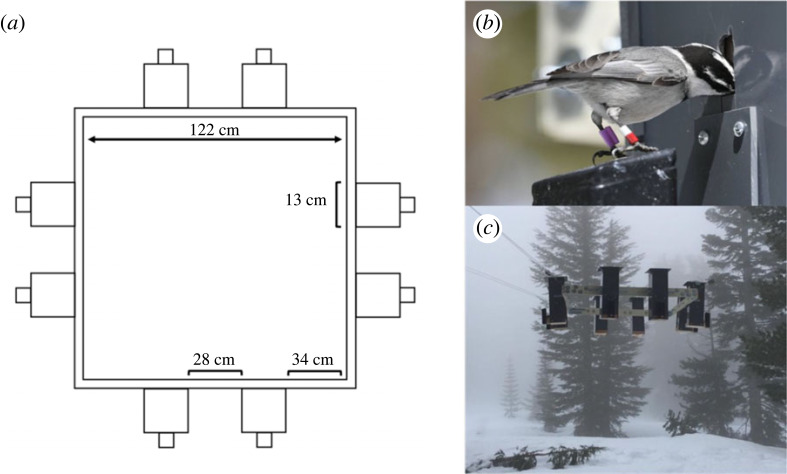
Schematic and photographs of spatial cognitive testing apparatus. (*a*) Aerial view of the testing apparatus depicting equidistant placement of eight ‘smart’ feeders. Each elevation site has two arrays. (*b*) A PIT-tagged mountain chickadee obtaining a sunflower seed from our ‘smart’ feeder. The purple band on the left leg of the bird is the PIT tag. (*c*) One of the arrays deployed at our high-elevation site.

Our sample size for this study comes from six years of spatial cognitive testing in wild, free-living birds (2017–2022). Each year, birds consistently visiting the feeder array were tested on two spatial cognitive tasks; a spatial learning and memory task and a single spatial reversal learning task. Birds are tested on both cognitive tasks using ‘smart’ feeder arrays established at each elevation (figure 1; see detailed feeder description in [16,29,42]). Each elevation has two feeder arrays, approximately 1.5 km from the other, testing mostly non-overlapping birds. In each spatial array, eight feeders are fixed equidistant on a square aluminium frame (1.2 × 1.2 m) raised *ca* 4–5 m above the ground and equipped with RFID technology and a motorized door that can be programmed to provide or restrict access to food [16,29,42]. The feeders operate in three modes: 1) Open mode (recruiting birds to feeders), where the feeder doors are left open, and any bird can gain access to sunflower seeds. (ii) all mode (habituation to feeder door movement), where the feeder doors are all closed, but will open when any PIT-tagged bird lands on the perch of any feeder; and (iii) target mode (testing cognitive performance), where feeders are programmed to only allow access to certain birds. In target mode, individual birds that have been actively visiting the feeder arrays during all mode are assigned to one feeder within the array and only when the bird perches on the antenna of its assigned feeder will the feeder door open and provide access to food. In all three modes, feeders recorded unique ID, date and time of visit, allowing us to assess feeder preferences (used in feeder assignments). For feeder assignments, each bird was assigned individually to one of the eight feeders at an array, such that we avoided a bird's preferred feeder. For birds participating in the task in multiple years, we chose a different feeder assignment in subsequent years. For the single spatial reversal learning task, all birds were re-assigned, pseudorandomly to a new feeder within the array [29,42]. Individuals assigned to the same feeder in the initial spatial learning and memory task were assigned to different feeders for the single reversal task to minimize effects of social learning.

Birds participate in these cognitive tasks on their own volition. Cognitive testing starts with 4 consecutive days of the spatial learning and memory task (using target mode described above), then at the start of Day 5 the assigned feeder for each bird is switched or ‘reversed’ and the birds are tested for an additional 4 consecutive days (8 total days of testing). We run each task for 4 consecutive days to maximize our sample sizes; however, an individual may not participate in every day of testing. For instance, a bird may come to the feeder during the spatial learning and memory task on Day 2, not Day 1. As such, we use the number of trials the birds complete on both tasks to assess performance. A trial began during target mode, when a bird visited any feeder within the array and ended when they visited their assigned feeder, with the subsequent visit starting a new trial. The number of non-rewarding feeders visited prior to visiting the correct rewarding feeder was considered the number of location errors [29].

We do not use any specific performance criteria to switch birds from the spatial learning and memory task to the single reversal task. However, only birds that completed at least 20 trials and made an average less than or equal to 3 location errors on both tasks were included in the analyses, following our standard protocol [22,28,29,42,43]. There are two reasons to use 20 trials cut-off: first, we used the mean number of location errors per trial over the first 20 trials as the metric of cognitive performance, so all birds have exactly the same experience of 20 trials, and second, 20 trials is sufficient for birds to learn the task [28,29,42]. For both spatial and reversal-learning performance, we calculated the mean number of location errors per trial (maximum of seven, because there are eight feeders in an array) a bird made across the first 20 trials and across the total trials completed during the 4-day testing period entire task. Although the mean number of location errors per trial over the first 20 trials and across total trials completed during the 4-day testing period are strongly correlated, we have included both in our analyses as they provide different information. Performance on the first 20 trials provides a measure of learning acquisition, while performance over the entire testing period provides a measure of longer-term memory and retention [22]. These performance measures have strong ecological relevance and have been shown to predict survival at high elevation [22], female reproductive investment at both elevations [40,41], and individual variation in these measures was associated with genetic differences [23]. In addition, we have previously shown that male and female chickadees do not differ significantly in their mean spatial or reversal cognitive performance [39] and that birds do not perform significantly better on the task in subsequent years (i.e. with experience) [22,42]. In all analyses, we use spatial cognition data from the first year a bird was tested, regardless of the year they bred. All social pairs compared were unique, meaning that the same bird may have been included more than once in our analysis, but only if they had paired with a new tested social mate.

### (c) Food caching propensity

We used the total number of trials completed throughout the spatial learning and memory task as a measure of food caching propensity. Chickadees complete hundreds of trials in a day and collect one sunflower seed during each trial. However, they can only eat a small fraction of seeds they collect from the feeders and cache most of the seeds they receive [44]. Food consumption probably varies much less than the individual variation in the total number of trials and hence the total number of trials appears to be a good metric for food caching propensity. In addition, individuals exhibit repeatable performance in the total trials they complete across years [36].

### (d) Monitoring breeding pairs

Across both elevations we maintain and monitor approximately 350 nest-boxes, which result in *ca* 50 nests a year at each elevation [45]. Nest-boxes are checked weekly for presence of nesting material, active nests are closely monitored for first egg date, clutch size and hatching date, and nestlings are weighed at day 16 (brood size). Across breeding stages, we identify parent IDs and assign sex based on behavioural and morphological traits (e.g. males sing in response to playback and have cloacal protuberances, females incubate eggs and have brood patches). Since 2019, we have equipped occupied nest-boxes with RFID readers to further confirm male and female IDs—visitation rates can be used to differentiate males versus females, as females incubate eggs and enter the nest-box far more often than males prior to hatching.

### (e) Statistical analysis

To be included in the statistical analyses on both tasks, an individual must have completed a minimum of 20 trials with a mean number of location errors less than or equal to 3 over the entire 4-day testing period. We tested whether social mate choice is associated with cognitive performance on the spatial learning and memory task using two approaches. First, we tested for assortativity in social pairs by testing for a correlation between male and female performance on the spatial learning and memory task. We ran separate linear models for each elevation, using the female's score (mean number of location errors per trial) as the response variable and the male's score as a fixed effect. Second, we tested whether females (or males) select social mates based on their spatial cognitive abilities by comparing spatial learning and memory performance scores within pairs. As our previous research shows that males and females do not differ in spatial cognitive performance [39], any observed differences within mated pairs are probably driven by mate choice. We ran separate linear models for each elevation with an individual's score as the response variable, sex and number of trials completed as fixed effects, and pair identity as a random effect to constrain comparisons within breeding pairs. Elevations were run separately because our previous work shows significant differences among birds inhabiting high versus low elevations (e.g. [16]). Each unique male–female pair was represented only once in all analyses, even if the pair bred together in multiple years.

We used the same approach to test whether reversal performance (a proxy for cognitive flexibility) or caching propensity (total trials completed) are associated with social mate choice. Analyses of reversal performance matched the models described above, only we used performance score on the reversal learning test as the response variable and as the fixed effect in the correlational approach. For analyses of caching propensity, we used total trials as a response variable (on the spatial learning and memory task only) and included sex as a fixed effect in the differences within pairs (second) approach. For both cognitive tests (spatial learning and memory, and spatial reversal learning) we conducted analyses on performance in the first 20 trials completed and across the total testing period.

To test whether divorce occurs when an individual has an opportunity to pair with an individual of higher cognitive ability, we compared the cognitive performance of the new partner with the previous partner (old partner). We ran the same analysis from the male and female perspective across new pairings that resulted from death versus divorce. We tested whether new and old partners differed in cognitive performance and total trials completed by running separate linear models for pair switches due to death versus those due to divorce. We included cognitive performance or total trials completed as the response variable, new versus old partner as a fixed effect, and focal partner identity as a random effect to constrain comparisons within an individual focal bird. We tested whether the differences between new and old partners differed across elevations by including interaction between partner (new versus old) and elevation in the model, but none of these interactions were significant (*p* > 0.05) so we report results from models without the interaction. For all models, we ran linear and linear mixed models in R v. 4.1.1 [46,47], and tested the residual fit of all models using the R package DHARMa [48].

## 3. Results

Using eight breeding seasons and 6 years of spatial cognitive testing in the wild, we were able to make male–female comparisons within social pairs for 76 pairs at high elevation and 56 pairs at low elevation for spatial learning and memory performance. For reversal learning performance, we were able to compare within 70 pairs at high elevation and 37 pairs at low elevation. As a proxy for caching propensity, we compared the total number of trials males and females completed in the spatial learning and memory task (following [36]), which allowed us to compare caching propensity within 76 pairs at high elevation and 56 pairs at low elevation.

### (a) Spatial learning and memory

Using mean performance across the first 20 trials completed for the spatial learning and memory task (mean number of location errors per trial), we found no significant correlation between male and female social partners at high elevation (*F*_1,74_ = 1.36, *p* = 0.247, adj. *R*^2^ < 0.01) or at low elevation (*F*_1,54_ = 2.02, *p* = 0.161, adj. *R*^2^ = 0.02) (electronic supplementary material, figure S1).

**Figure 2.**
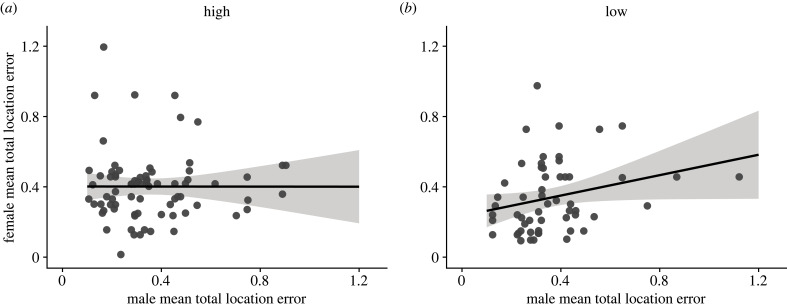
Mean performance of male and female pairs across total trials completed on the spatial learning and memory task at (*a*) high and (*b*) low elevations. Social mates' spatial learning and memory performance approached statistical significance at low elevation (*b*), but not at high elevation (*a*).

When we compared male and female social partners' performance across total trials completed during testing, there was no significant correlation at high elevation (*F*_1,74_ < 0.01, *p* = 0.993, adj. *R*^2^ = −0.01); however, at low elevation the positive correlation between male and female partners’ performance approached significance (*F*_1,54_ = 3.95, *p* = 0.052, adj. *R*^2^ = 0.05) (figure 2).

We also assessed whether performance on the spatial learning and memory task differed between males and females within a social pair. Across the first 20 trials completed, there was no significant difference between mated males and females at high elevation (*F*_1,150_ = 1.63, *p* = 0.203) or at low elevation (*F*_1,55_ = 0.34, *p* = 0.561). We found a similar pattern across total trials completed: mated males and females did not differ in their performance at high elevation (*F*_1,77.3_ = 0.03, *p* = 0.854) or at low elevation (*F*_1,55.4_ = 0.05, *p* = 0.827).

### (b) Reversal spatial learning

Using mean performance across the first 20 trials completed for the reversal spatial learning task, we found no significant correlation between males and females within pairs at high elevation (*F*_1,68_ = 0.31, *p* = 0.581, adj. R^2^ = −0.01) or at low elevation (*F*_1,35_ = 0.11, *p* = 0.739, adj. *R*^2^ = −0.03).

Similarly, across total trials completed during testing, we found no significant correlation between male and female social partners' reversal learning performance at high elevation (*F*_1,68_ < 0.01, *p* = 0.952, adj. *R*^2^ = −0.01) or at low elevation (*F*_1,35_ = 0.54, *p* = 0.468, adj. *R*^2^ = −0.01) (electronic supplementary material, figure S2).

**Figure 3.**
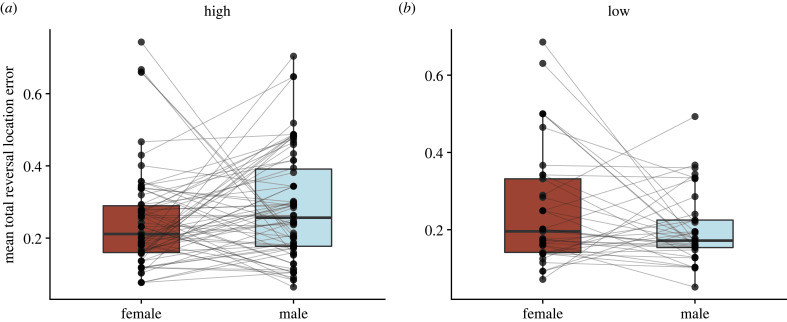
Mean reversal learning performance across total testing period for socially mated males and females at (*a*) high and (*b*) low elevations. Females tend to make fewer errors on the reversal learning task than their social males at high elevation (*a*), but do not differ at low elevation (*b*). Lines connect socially mated male–female pairs. Boxplots show median and first and third quartiles.

We also assessed whether performance on the reversal spatial learning task differed among males and females within a social pair. Across the first 20 trials completed, females tended to perform better than their social males at high elevation (*F*_1,138_ = 3.56, *p* = 0.061); however, there was no significant difference between males and females at low elevation (*F*_1,72_ = 0.85, *p* = 0.359). We found a similar pattern across total trials completed: females tended to perform better than their males at high elevation (*F*_1,138_ = 5.80, *p* = 0.059); however, there was no significant difference between males and females at low elevation (*F*_1,72_ = 7.28, *p* = 0.320) (figure 3).

### (c) Caching propensity

**Figure 4.**
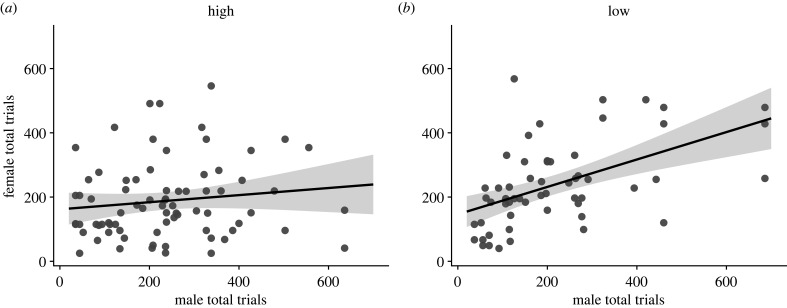
Total number of trials completed on the spatial learning and memory task within male and female social pairs. Social mates exhibit similar caching propensities at low elevation (*b*), but not at high (*a*).

**Figure 5.**
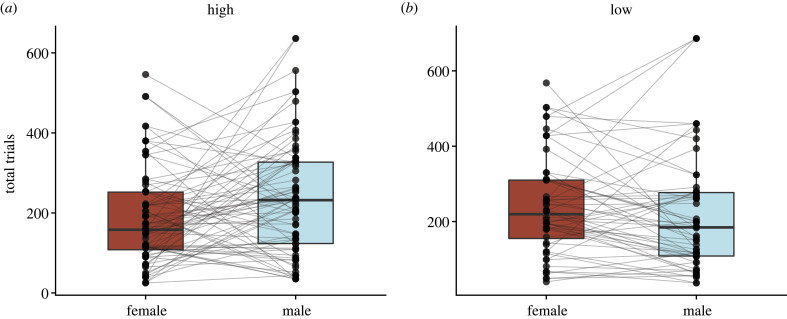
Comparing total number of trials completed on the spatial learning and memory task by socially paired males and females. Within pairs, males complete more trials than their social females at high elevation (*a*), but not at low (*b*). Lines connect socially mated male–female pairs, and boxplots show median and first and third quartiles.

As a proximate estimate of individual caching propensity, we compared the total number of trials completed over 4 days of the spatial learning and memory task within male and female social pairs. We found no correlation between the total number of trials completed by males and females within pairs at high elevation (*F*_1,74_ = 1.32, *p* = 0.254, adj. *R*^2^ < 0.01); however, at low elevation the total number of trials completed by males and females was significantly positively correlated (*F*_1,54_ = 20.63, *p* < 0.001, adj. *R*^2^ = 0.26) (figure 4). Within pairs, males completed significantly more trials than females at high elevation (*F*_1,75_ = 5.77, *p* = 0.019), but not at low elevation (*F*_1,55_ = 1.14, *p* = 0.291) (figure 5). However, when we compared the total number of trials completed by males and females at high and low elevations, regardless of who they were paired with, males and females completed similar numbers of trials at both high elevation (*N* = 186, *F*_1,184_ = 1.22, *p* = 0.272) and low elevations (*N* = 121, *F*_1,119_ < 0.01, *p* = 0.926).

### (d) New social mate pairings

Social pairings may change from year to year, due to death of a mate or divorce. We considered a social pairing switch to be a divorce if the previous partner was detected alive and breeding with another individual or if the partner did not breed with the focal bird but was detected alive during the following autumn/winter testing season. If the partner was not detected, we considered it to have died. Using these categories, we conducted separate analyses to compare performance of new and old partners resulting from death versus divorce. We conducted analyses separately for males and females, because our dataset is made up of male–female social pairs. Elevation was included in the original model, but there was no effect of elevation, so it was removed from analyses and high and low elevation birds were combined to bolster sample sizes.

Among females, there were no significant differences in spatial learning and memory performance between old and new mates resulting from divorce (*N* = 23 comparisons, first 20 trials: *F*_1,22_ = 0.02, *p* = 0.892; total trials: *F*_1,22_ = 1.17, *p* = 0.291) or from death (*N* = 25 comparisons, first 20 trials: *F*_1,24_ = 2.85, *p* = 0.104; total trials: *F*_1,24_ = 0.07, *p* = 0.800). We found similar patterns for reversal spatial learning performance; old and new mates resulting from divorce did not differ in performance (*N* = 19 comparisons, first 20 trials: *F*_1,36_ = 0.32, *p* = 0.577; total trials: *F*_1,18_ = 0.08, *p* = 0.776), nor did old and new mates resulting from death (*N* = 17 comparisons, first 20 trials: *F*_1,16_ = 0.78, *p* = 0.389; total trials: *F*_1,16_ = 1.55, *p* = 0.231).

Among males, we found similar patterns, none of the above comparisons of old and new female partners differed significantly in their spatial or reversal learning performances regardless of whether the new pairing was due to death or divorce (all *p* > 0.05, electronic supplementary material, table S1).

We conducted further analyses to compare the total number of trials completed among old and new social partners resulting from death and divorce (for males and females separately as well), and again found no significant differences among old and new partners regardless of category (all *p* > 0.05, electronic supplementary material, table S2).

## 4. Discussion

We found weak evidence for the hypothesis that spatial cognitive abilities and caching propensity influence social mate pairings. Performance on the spatial learning and memory task weakly correlated within male and female pairs at low elevation, but not at high elevation, and we found no significant difference in performance between paired males and females at high or low elevation. Furthermore, we found no correlation between paired males and females in their reversal spatial learning performance, a proxy for cognitive flexibility, at either elevation. However, females tended to perform better than their social males at high elevation, while there was no difference between paired males and females at low elevation. Our results suggest that females and males may pair assortatively based on spatial cognitive abilities, but only under certain environmental conditions and only for some aspects of cognitive performance. For instance, the comparison of socially paired male and female cognitive abilities suggests that males may seek out cognitively flexible females when choosing a social mate, but perhaps only at high elevations. These relationships are interesting given what we know about the functions of spatial versus reversal learning and the population level differences in performance on these two cognitive tasks: high-elevation birds perform better on the spatial cognitive task, while low-elevation birds perform better on the reversal learning task [16,27–29,42].

Between elevations, selection pressures on spatial cognitive abilities generated by overwinter survival may differ from those driven by mate choice. Natural selection acts more strongly on overwinter survival and associated spatial learning and memory abilities at high elevations compared with low elevations [40,41,49], and we have hypothesized that there is a trade-off between spatial learning and memory ability and reversal-learning performance (proxy for cognitive flexibility) [27,29]. More recent work further supports this trade-off, as low-elevation mountain chickadees performed significantly better than high-elevation birds on a serial spatial reversal task [28]. Based on our findings, it is possible that natural selection acts more strongly on spatial learning and memory at high elevations and acts more strongly on reversal learning, or cognitive flexibility, at low elevations. These hypothesized differences in the strength of selection on cognitive traits may leave little variation available for sexual selection, leading to the associations we found between cognitive performance and social mate choice at each elevation. Specifically, if natural selection on spatial learning and memory is strong at high elevations, our finding of no relationship between spatial learning and memory and social mate choice at high elevations may be expected, as there may be limited standing variation. Similarly, if natural selection on cognitive flexibility is strong at low elevations, our finding of no relationship between cognitive flexibility and social mate choice at low elevations may also be expected, as natural selection may leave little standing variation in reversal learning performance. That said, while neither of these trends were statistically significant, the nature of the relationships between spatial cognitive performance and social mate choice differed among birds at high and low elevations; spatial learning and memory performance weakly correlated within pairs at low elevation, while reversal learning performance did not correlate within pairs at high elevations.

In this study, we used data from a single reversal to assess cognitive flexibility, which tests how fast a bird learns a new association while unlearning or extinguishing the response to the previous stimulus. As a bird learns the reversal task, it should stop visiting the previously rewarding feeder or any other feeders within the array. While our reversal learning task is spatial, performance on this task is not directly affected by spatial learning ability, as we see opposite relationships between performance on the initial spatial learning and memory task compared with the reversal task [28]. Therefore, this reversal task taps into additional learning and memory ability probably associated with inhibition and executive control [50,51].

While individual variation in spatial learning and memory ability is associated with direct fitness consequences [22] and has a genetic basis [23], it is not clear whether differences in reversal learning ability are heritable, as we previously found no association between reversal learning performance and overwinter survival [22]. We have previously argued that reversal ability may be directly affected by memory load, with higher memory load providing higher proactive interference which can negatively affect reversal learning via perseveration on the original rewarded response [27]. Thus, selecting mates with better spatial learning and memory abilities can have direct effects on both mates, as they would produce young with better spatial learning and memory abilities; however, it is not clear what or if any fitness benefits may be incurred by selecting mates with better or worse reversal learning abilities.

Previous work assessing caching propensity in the field and in a common garden experiment has shown that chickadees inhabiting harsher environments, including higher elevations and latitudes, cache more food items compared with those inhabiting milder environments, suggesting that caching propensity is heritable [34–36]. Because caching is critical for overwinter survival and is likely heritable, caching propensity may be relevant for social pairing decisions. We found that caching propensity, estimated by total trials completed on the spatial learning and memory task, significantly correlated within social pairs at low elevation; however, within pairs at high elevation, males cached significantly more food than their social females. Work in the closely related black-capped chickadee suggests that males provide a buffer for females against the harsh winter climate by allowing them access to their food stores [52]. Since chickadees form social pairs within winter flocks [53], females may choose social males that cache more than themselves to increase their likelihood of survival, especially at high elevation where winters are comparatively harsher. In addition, such pairing decisions would result in offspring with higher food caching propensity leading to higher fitness due to increased offspring survival. At low elevation, it is possible that the correlation within pairs is simply a result of males and females visiting the feeders together; however, we do not think this is the case, as the total number of trials used to measure caching propensity is repeatable within individuals at our study site, regardless of who they are paired with [36]. In addition, we found no overall differences in the total number of trials between males and females at both elevations, yet within mated pairs at high elevation, males completed significantly more trials than their mates. Such differences suggest that females choose to pair with males that cache at similar rates or better than themselves when provided the option.

An important consideration when interpreting these results is the reliance on social information. Heinen *et al*. [43,54] found that high-elevation birds rely less on social information when discovering novel food locations compared with low-elevation birds. If high-elevation birds generally rely less on social information compared with low-elevation birds, we might expect them to rely less on social information provided by their social mates. For instance, we have shown that higher reproductive output in our system is associated with female breeding experience and not with pair longevity or influence of social mate [55]. Furthermore, since winter conditions are much harsher at high elevations compared with low elevations, birds may have little time to spend assessing the cognitive abilities of social mates. Any bird that survives the harsh winter and into the breeding season probably has sufficient spatial cognitive abilities, resulting in the cost of social mate assessment outweighing the marginal benefit for high-elevation birds.

Our predictions for social mate assessment are based on previous research showing that females can discriminate among males with better and worse spatial cognitive abilities [40,41,56]; however, the mechanism for this discrimination remains unknown. Females may assess males’ spatial cognitive prowess via direct observation of caching and retrieving food items, general body condition, or secondary sexual traits. Chickadees are socially paired within their autumn/winter flocks [53], providing an opportunity to directly observe males. However, direct observation of fitness traits is generally thought to be time consuming and rare, while secondary sexual traits provide information indirectly, but almost instantaneously [18]. If females are using a secondary sexual trait as a cue for mating decisions and the cue and trait are mismatched, we may not see strong effects of social mate preference, which could explain the rather weak relationship we see between social mate choice and spatial cognitive abilities and caching propensity. Furthermore, while 8 years of breeding data is substantial in the field of animal behaviour, it is still only a snapshot in evolutionary time, and more time may be necessary for a purported cue to become a true communicative signal.

Although mate preference and choice are often equated in the literature, social mate choice is probably limited in the wild, resulting in pairings that are ‘suboptimal’. This may be particularly relevant in chickadees, as they form social pairs within their overwinter flocks and are highly sedentary [53]. As a result, most of the male–female pairs within a flock have carried over from the previous year, leaving juveniles a limited set of social mates to choose from when they join a flock post-fledging. The additional analyses we conducted comparing new pair formations resulting from death versus divorce provide further information on pairing decisions. If cognitive performance or caching propensity were important for social mating decisions, we would expect individuals that divorced and re-paired with a new individual to choose a new social mate that exhibits better performance and caches more food, but this was not the case. We saw no differences between the performance of old and new social partners regardless of whether the new pairing was a result of death or divorce. That said, these analyses suffer from comparatively smaller sample sizes, and we have no information on who initiated the divorce in each of these instances [57].

In conclusion, we found weak evidence that spatial cognitive performance and caching propensity influence social mating decisions in mountain chickadees, and the relationships we found between spatial and reversal cognitive performance, caching propensity, social pairs and elevation were not as we predicted. Given the likely limited availability of unpaired mates, it is possible that extra-pair mating choices are more relevant to differences in cognitive abilities and propensity to cache food than social pairing. We are currently working to assess extra-pair mate choices in our system, to determine whether females seek extra-pair males that perform better on our spatial cognitive tasks and exhibit a higher caching propensity. Moving forward, research aimed at understanding mating decisions should consider both social and genetic mate choice, particularly among species that exhibit biparental care.

## Data Availability

Raw data and code are available via Dryad, https://doi.org/10.5061/dryad.g79cnp5tv [58]. Supplementary material is available online [59].
